# Odontological Society of Pennsylvania

**Published:** 1897-11

**Authors:** 

**Affiliations:** Odontological Society of Pennsylvania


### ODONTOLOGICAL SOCIETY OF PENNSYLVANIA.

THE regular meeting of the Society was held March 13, 1897, the President, Dr. C. N. Peirce, in the chair.

Upon the completion of routine business, the President requested the essayist of the evening, Dr. N. V. Ball, to read his paper upon the subject, "The Mouth as the *Via Natura* for the Entrance of Disease."

(For Dr. Ball's paper, see page 724.)

DISCUSSION.

*The President.*—This interesting paper is before you for discussion. We shall be glad to hear from any member on the subject. Dr. Ball will be pleased to answer any questions that are suggested. I have been very much interested, indeed, in the paper, but I think that the doctor's lack of experience in dental operations has probably induced him to attach too much importance to wounds in the month as being open to infections for disease. When we consider the thousands of teeth that are extracted, the instances of serious results are rare. We do have some very serious conditions following extractions, but I think it is rather remarkable that they seldom occur, and this would indicate that the wound from the removal of a tooth was not a very serious matter.

While nearly every dentist applies some antiseptic at once, simply to satisfy the patient's mind,—and probably it is a good precaution, in a measure,—yet there are many teeth extracted where that is not done, and yet rarely do we have inflammation following the extraction. The instances where it occurs are in the removal of a third molar, between the second molar and the jaw, and where inflammation was present previous to the extraction. Indeed, I think that in every instance where inflammation follows extraction, it existed in the pericemental membrane previous to the extraction. Those instances do give the patients severe suffering.

Then regarding the effect of dentition upon the infant. The doctor explains a question that I was asked the other day when speaking to students about lancing the gum in cases of retarded eruption of the teeth. They said to me, "Are you not afraid of infection by lancing the gum?" I said I had never recognized it as a dangerous operation. Where we have what we call interrupted dentition, the source of trouble is from the resistance the gum offers to the advancement of the crown of the tooth, and that forces the tooth back into the uncalcified portion back of the pulp. And if the resistance offered by the gum is sufficient to prevent the advancement of the crown, it produces inflammation at the base and through the fifth pair, and wherever the filaments of those nerves may be distributed.

In regard to the allusions made to pneumonia during dentition. I have several families under my care, and in one family every child, previous to the eruption of the teeth, has been taken with severe catarrhal affections, bordering on pneumonia. I have lanced the gum freely, and the catarrhal affection subsided at once, showing that the symptoms were from the irritation of the nerve caused by the crown pressing back on the uncalcified portion. That has occurred so often that I am satisfied that the tendency to catarrhal affection is induced by that irritation, and relieved just as soon as the pressure on the crowns of those teeth is removed, relieving the pressure at the base.

Lancing the gums is a favorite practice with me. I advise it in every case where the children are nervous. Never yet, of the dozens, if not hundreds, of children's mouths I have lanced, have I seen a single case of irritation following the lancing; but in almost every case I have seen a subsiding of the disturbance by the operation. So I am an advocate, a strong advocate, of the lance in eruption of the teeth. There have been children that recognized during eruption of their teeth the comfort the lance has given them, and they submitted to it with pleasure.

Notwithstanding I have made these little criticisms, I can readily see that there may be cases where we should be cautious and use antiseptic treatment where serious wounds have been made in the mouth, as in case of laceration of the pericemental membrane.

*Dr. Head.*—I was very much interested in this paper, and it brings up a great deal of material that will be of value to all dentists if they will apply it, not only in a general but special way, to their practice.

It is, as Dr. Peirce has said, a cause of wonder that wounds do heal so quickly in the mouth. But if they did not, it seems to me that it would be next to impossible for us to apply real antisepsis in that region, for while we might apply a mouth-wash once or twice, or every hour in the day, still the germs would grow and contamination would take place. And if there could be any way by which wounds in the mouth could be treated with the same antiseptic care that wounds are treated on the arm or leg, I think that all dentists would consider that a great boon had been given the dental profession.

*Dr. Faught.*—I have listened with a great deal of interest to this paper of Dr. Ball's. I stand, in the dental profession, for the maintenance of an antiseptic condition in the treatment of teeth.

I know that there is a disposition on the part of many to cavil at the possibility of infection, or at the needless expenditure of time and effort to overcome the thousand and one germs which are constantly getting the better of our efforts. But the citing of cases of patients that do not become infected, I do not think relieves us in this age from applying the known means of combating germ troubles.

I am glad that Dr. Ball says the open wound is liable to very serious dangers. For the production of these troubles there are always three conditions—the germs, the lesions, and, still further, the pabulum—for these germs to grow and multiply upon. Now, where any one of these conditions is lacking, the infection is lacking. But we are so liable to have these conditions come together, and where we do have the trouble we have it just because the condition predisposes to the life of these germs. Therefore I believe we ought in all of our operations to be careful not only to instil into the minds of our patients the importance of this matter, but I believe that we ought also to use the utmost care of our instruments and everything we place in or about the mouth, and not carry the pathogenic germs from the mouth of one patient into that of another. That the probabilities in some cases are extremely strong there is no question, and we should use every effort to prevent the transfer of these germs.

I remember a gentleman presented on one occasion—I do not remember whether it was at a meeting or in private—a case of pyorrhœa alveolaris. He was speaking of the construction of an artificial denture, and told of a patient who persisted in the maintenance of a loose eye-tooth. He felt sure that if he could get that eye-tooth away, he could construct a denture. The patient persisted in refusing to part with that tooth. So, the story goes, he prepared an instrument from a case of pyorrhœa, and simply swept it around the neck of that tooth, with the result that the patient lost the tooth. The tooth extracted itself, as it appeared to her. That is perfectly possible, and shows that we should be extremely careful of the instruments.

Now, then, the great question arises as to whether the applications of antiseptics to an open wound are of no use, as Dr. Head says——

*Dr. Head.*—I did not say they are of no use. I said we could not apply them in the same manner as in other parts of the body.

*Dr. Faught.*—Well, the trend of your remarks——

*Dr. Head.*—I hope the President will restrain the gentleman from commenting on the "trend" of my remarks.

*Dr. Faught.*—Well, then, I will simply confine myself to the case in my mind, presented to my consideration to-day, in which some correction of irregularity had been performed.

The patient was a young girl, twenty-one years of age, or not more than twenty-five. In the construction of the appliance the operator carried into the mouth a band around the wisdom-tooth in some way. Concomitant with the insertion of that appliance, the patient applied to the operator, complaining of a serious ulcer, so she termed it, just at the posterior outer cusp of the wisdom tooth. The operator, supposing that, possibly, in the insertion of the appliance he had in some way pinched or wounded the tooth, looked upon it as unlikely to give further trouble.

At the end of three months *that ulcer is still there* and unhealed, and the gentleman has applied his applications to it in vain, and it still exists.

Now, whether it is some other infection that has produced that ulcer in the mouth, that would have occurred any way, I am not prepared to say. But the fact of its not healing, when it was apparently a simple ulcer, leads me to believe that, either owing to the impaired vitality of the patient, or the condition existing in her system at the present time, or a constant autoinfection going on there, that ulcer has not gone through the ordinary process of healing.

I was pleased to have Dr. Ball dwell upon that in his paper. So far as an application is concerned, I advise the application of strong tincture of iodine, to see if we could not bring back a healthy condition of tissue, placing the patient upon tincture of iron and some peptones—peptonoids—to build up the system at the same time in connection with it.

I would like to ask the doctor, too, if he would give some remarks regarding the possibility of infection from syphilitic troubles in the mouth,—as to which is the more troublesome, the primary or the secondary,—as to which condition would be an absolute debarrer, and as to which condition an operator might serve a patient. I would be very much interested in that portion of the possibilities. Because I think it is a question at the present day as to how far a person in practice has a right to take under his care a person in whom he recognizes a syphilitic condition. We do have those patients, and from good families. It is a question as to whether we ought to say to them, " We cannot do anything for you;" whether to debar them. How are we to meet the case?

*Dr. Head.*—I am sorry to have spoken so much this evening, but to make the "trend" of my remarks more clear, I wish to say that the only thing I had in mind was that in any ordinary surgical operation, if the knife is infected or not rendered thoroughly antiseptic, no matter how carefully you may wash afterwards, the formation of pus will take place. It seemed to me hardly possible that any wound in the mouth could be treated so that infection would not be allowed to take place in the intervals of antiseptic washings.

*The President.*—Are there any further remarks from the Society?

We shall be glad to hear from Dr. Ball in explanation of the questions.

*Dr. Ball.*—In regard to syphilis: I did not take up those diseases that were not produced by germs. My remarks this evening had special relation to bacteria and such diseases as are of microbic origin, because that is the special subject in which I am interested and upon which I have been working.

Of course, it has been long recognized that syphilis was a common cause of infection. It is very contagious, especially the syphilis about the mouth, in mucous patches, which can be giving by kissing from one person to another. A person can be contaminated by kissing a person who has a mucous patch on the mouth. If, however, there are no lesions, the disease cannot be communicated in that way. As a rule, all those cases in which it has occurred have been where there has been some particular lesion about the mouth. One case I remember, which a woman contracted from her son by drinking out of the same cup.

Dentists may become infected by the fingers coming in contact with sores about the mouth, and should be very careful in their operations.

In regard to the particular ulceration mentioned, it is a difficult matter to say just what was the cause of that, or how to treat it, without seeing it. I should say that treatment by silver nitrate would have been beneficial.

It is astonishing that there are not more abscesses occurring from extraction of the teeth in practice. I can recall several instances of these arising from extraction. The dentists are not so apt to see these cases as the surgeon. I think that while they are not common, they are of sufficient frequency to require that some precaution be taken. Cuts about the fingers are not unusual, and often go untreated, and yet we know that these may become seriously affected. A surgeon making a cut about the finger treats it antiseptically, although he knows that thousands of people cut their fingers every day and do not treat it at all.

In my paper I should have read that a wound on the extraction of a tooth should be treated as any other wound *in the mouth.* I read it as "any other wound." Of course, wounds in the mouth are difficult to keep clean, and yet if you are compelled to treat a wound there you do use precautions. Of such ordinary aseptic solutions as are given, probably the bichloride of mercury is most efficient.

The mouth can be washed out by rinsing after the use of food or at other times of the day. I think that while we cannot keep wounds in the mouth clean, as we cannot keep wounds of the bladder clean, or wounds about the rectum, or about any large cavities of the body which are exposed to the air and exposed to these infections, we can do the next best thing,—do the best we can.

We do not to-day recognize any catarrhal troubles as produced by irritation. We look upon all of them as produced by germs. Pneumonia is undoubtedly produced by a distinctive bacteria. Meningitis is produced by bacillus. I have practised largely among the children of the poor, and I find frequently that pneumonias happen during teething. Sometimes I think that probably the child is in a weakened condition at this time and more susceptible to disease. The more I look into it, the more I think that it is due to this local infection. I believe that the lancing may do good and relieve the irritation about the place. It may also relieve some local matter,—let out some of the local fluid that may be contained there. Of course, as a rule, there is no abscess, and we know that where there is no contagion the lancing often does a great deal of good. So in this case it may do good. We know that children do become infected from the milk, and have various disorders of the stomach from this source; why cannot infection occur through the inflamed condition of the gums? It is known now that the diarrhœal condition is produced by germs. The stomach is in an irritated condition from various causes, and then the germs normally present act rapidly. Diarrhœa is not produced by a specific bacillus of diarrhœa. It was formerly thought that there were certain bacteria that caused it; but to-day we know that many bacteria that are usually harmless do gain entrance into the body and so produce these diseases. And it is the same in the mouth. I do not know that I can say any more.

*The President.*—Are there any further remarks on this interesting subject?

*Dr. Broomell.*—Before the subject is passed, I would like to move that we extend a vote of thanks to Dr. Ball for his very interesting paper.

Motion carried.

*The President.*—The next business is incidents of practice.

*Dr. Voight.*—I would like to show the members a little novelty in the shape of a box for holding sand-paper disks. It is compact, and the disks are easily picked out. It is manufactured by the S. S. White Company.

JOSEPH HEAD, M.D., D.D.S.,
*Editor Odontological Society of Pennsylvania.*

